# Supplementary figures and images for: Lamins gate nuclear and chromatin structures for cardiomyocyte maturation genes
Source: bioRxiv. 2026 Jun 12:2026.05.20.726565. Originally published 2026 May 22. Preprint. [Version 2] doi: 10.64898/2026.05.20.726565 (PMC13228526; doi:10.64898/2026.05.20.726565)

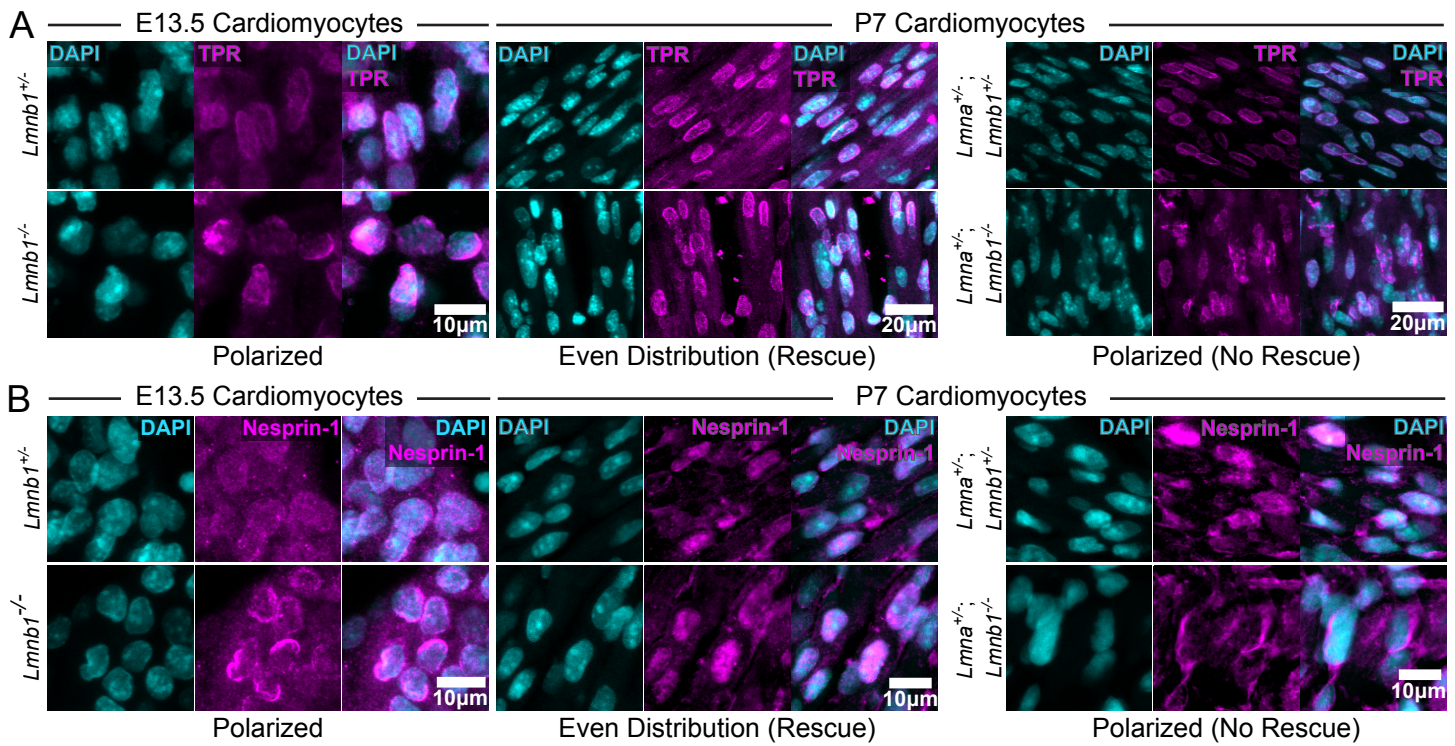

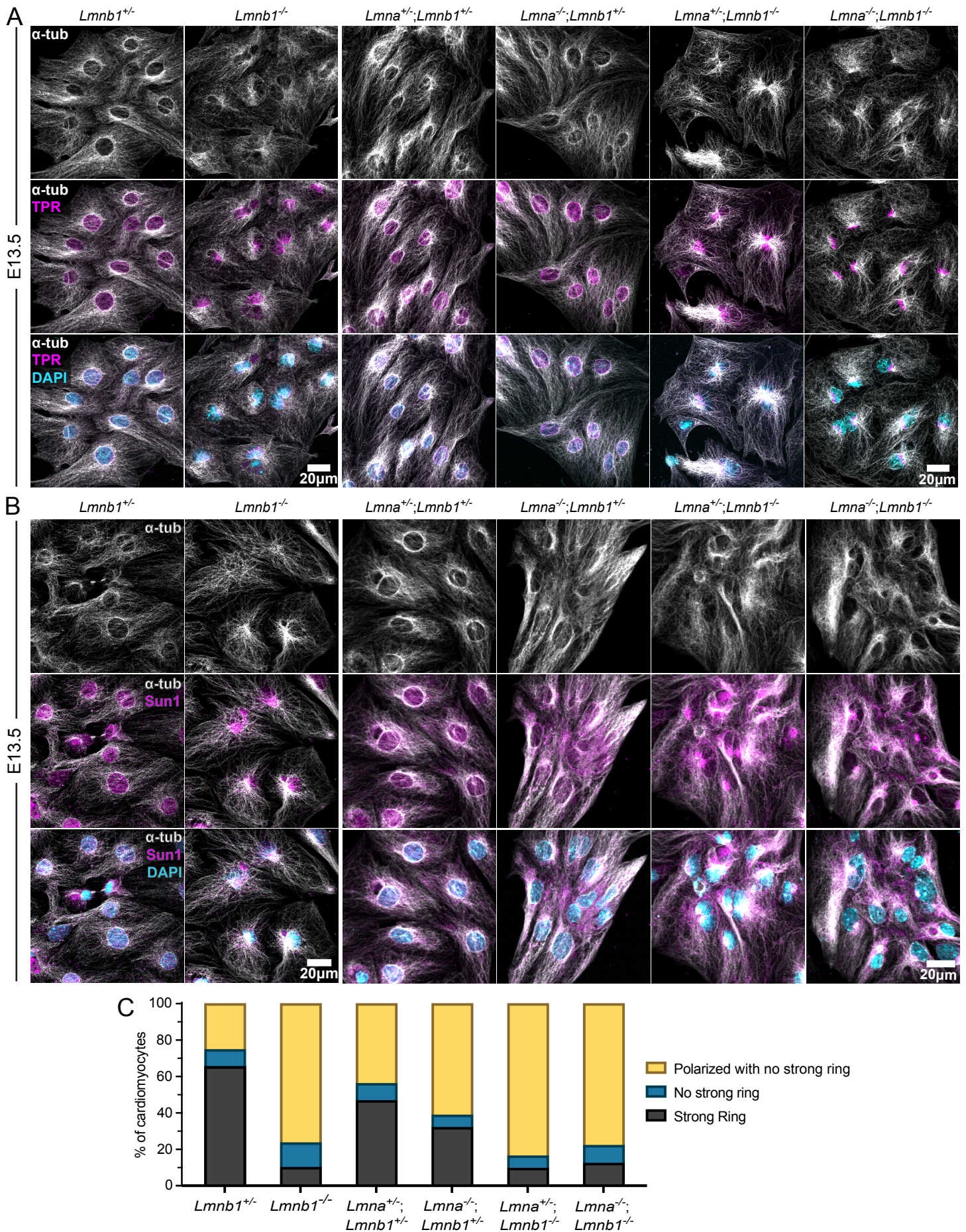

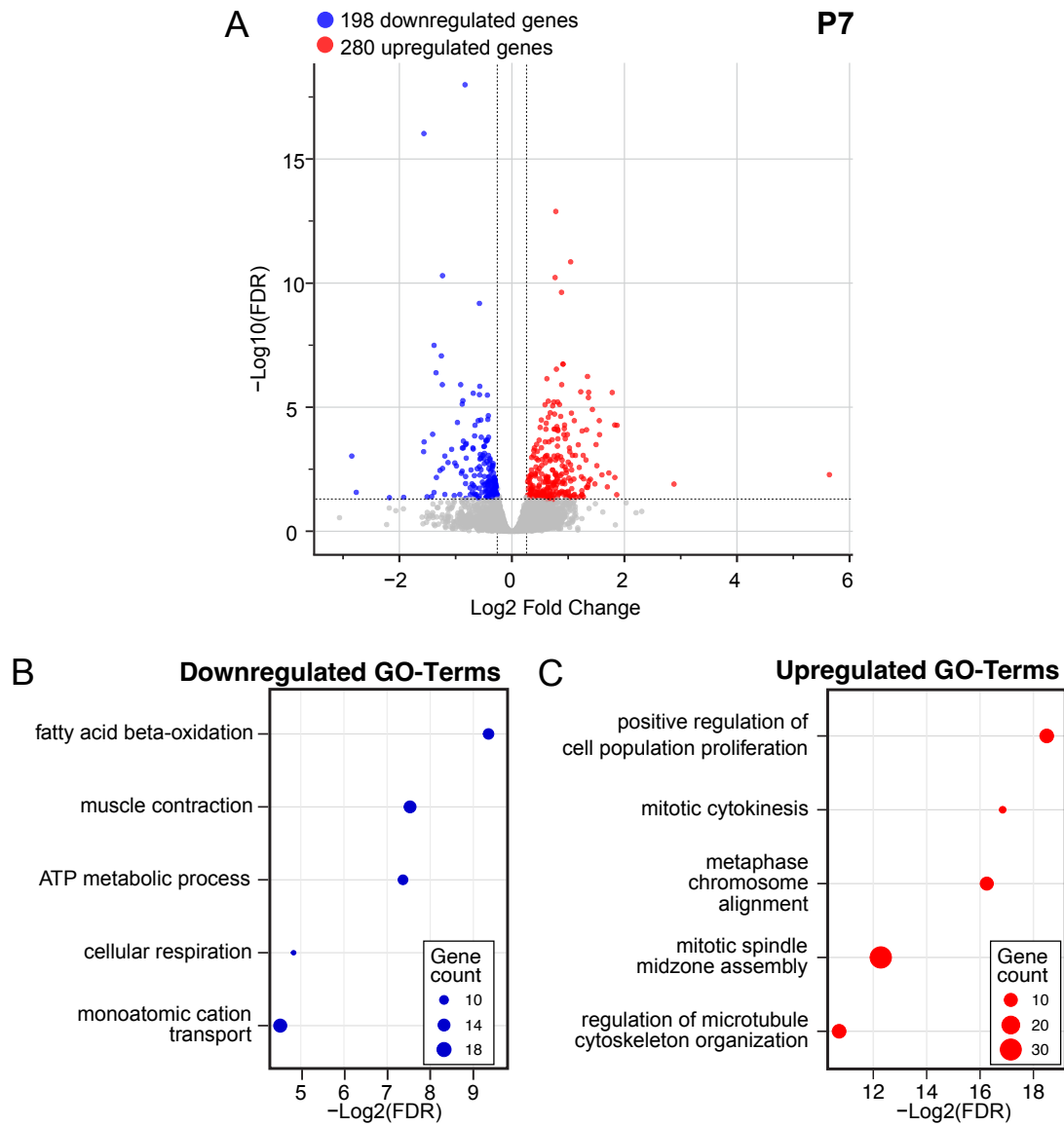

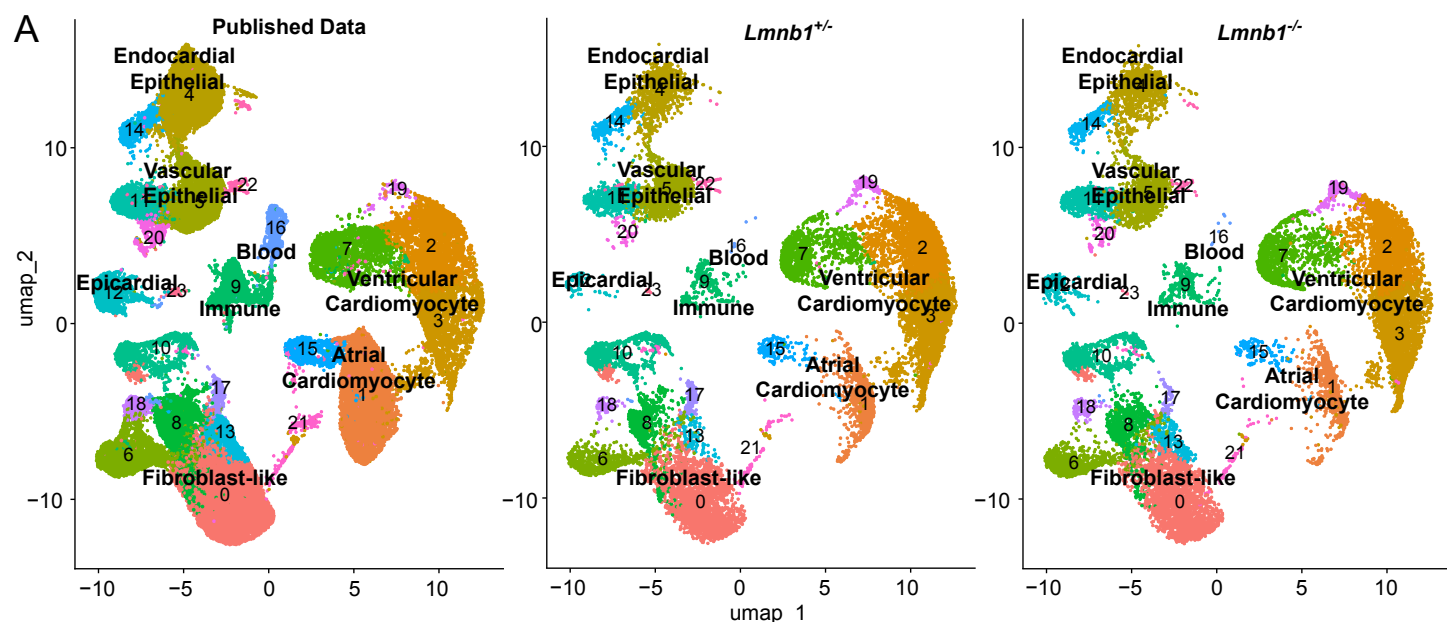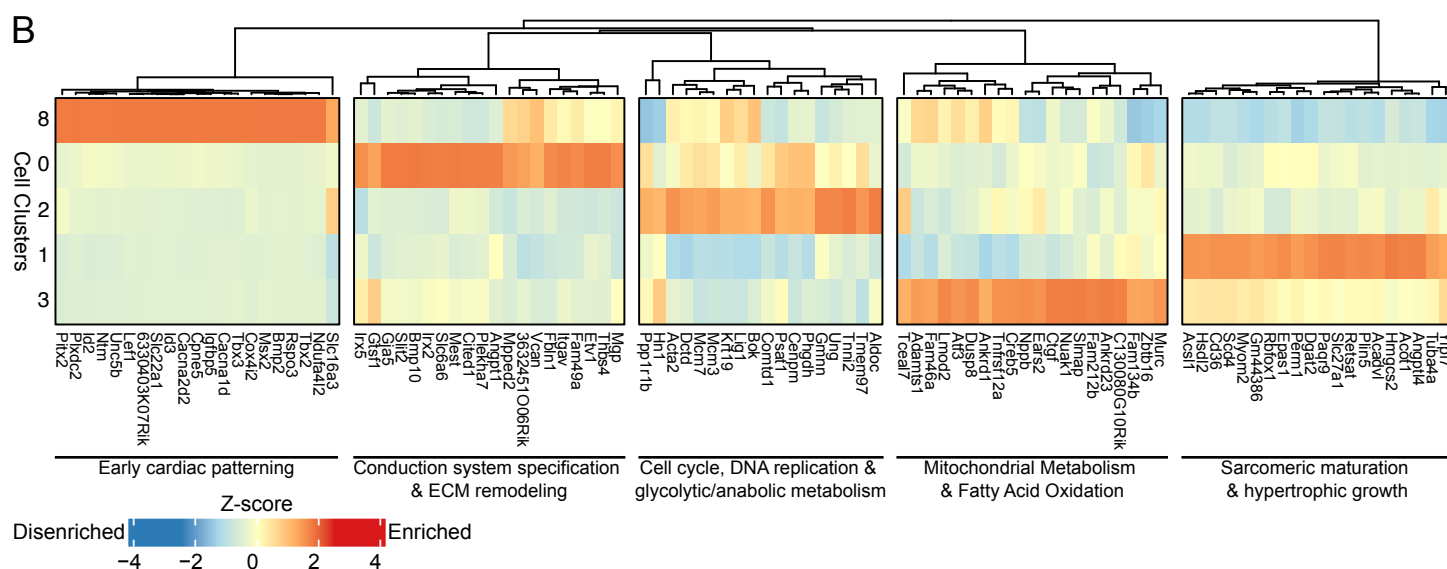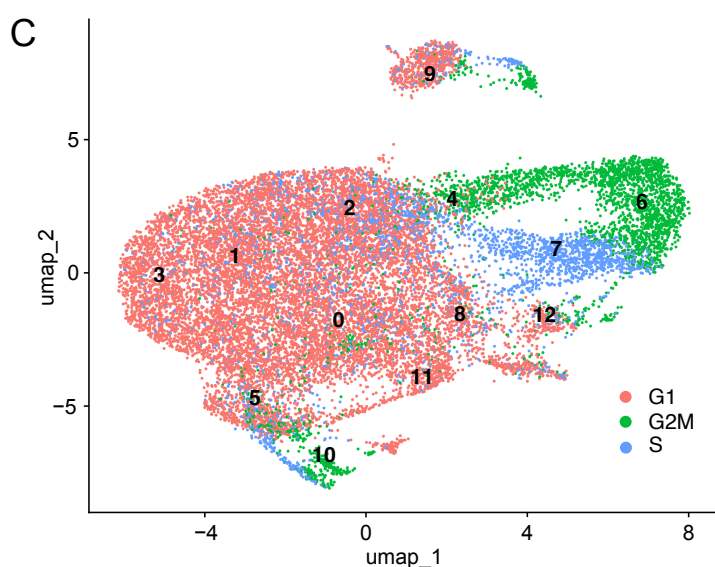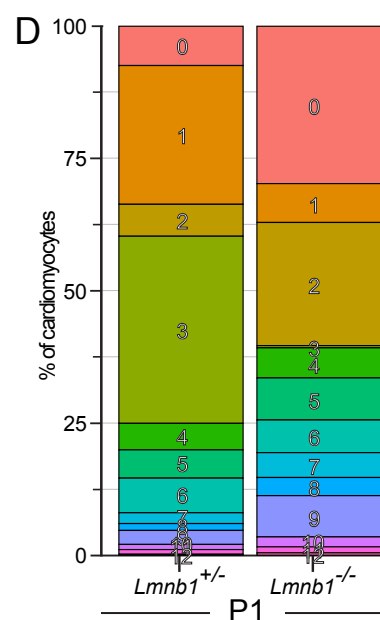

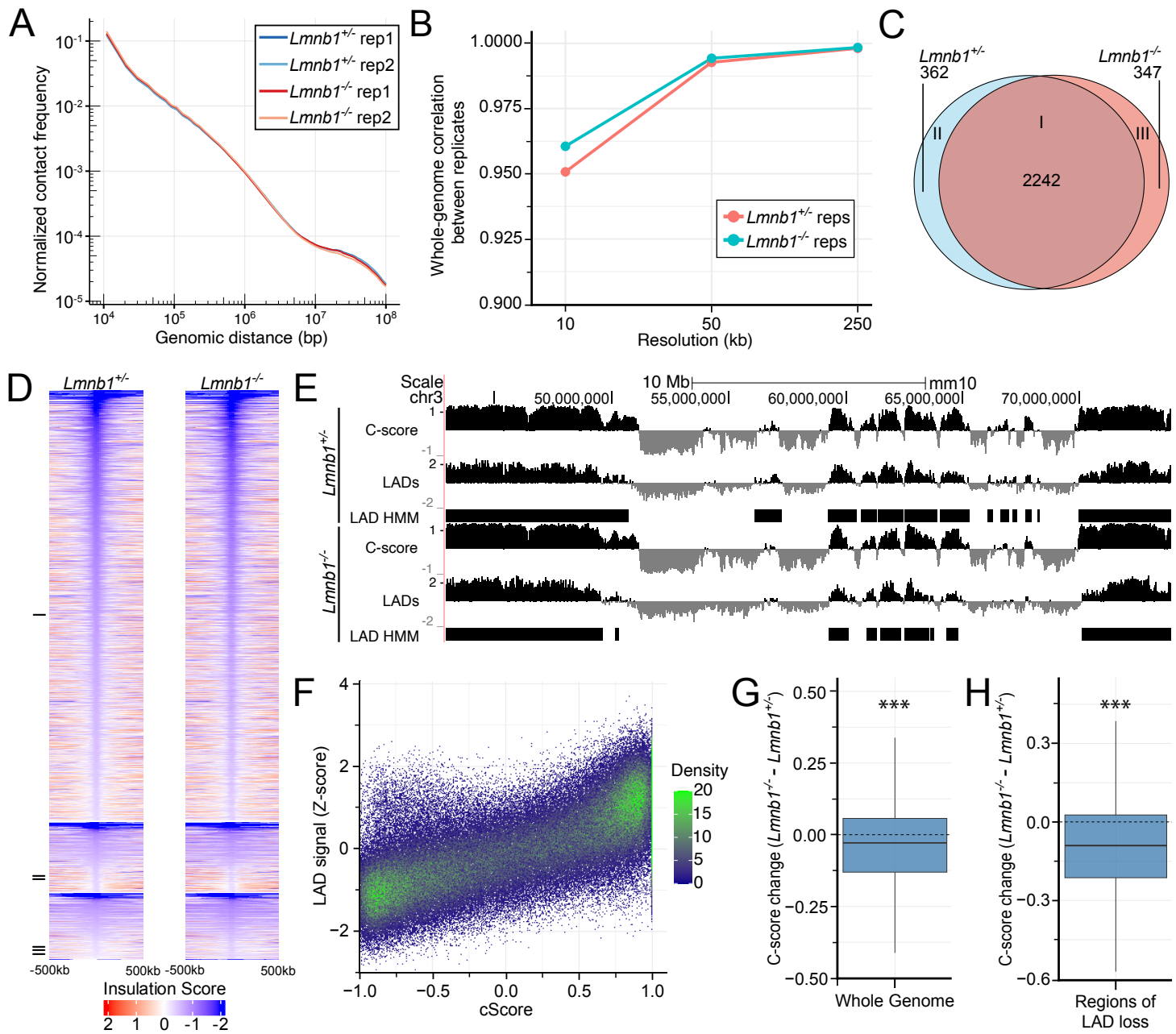

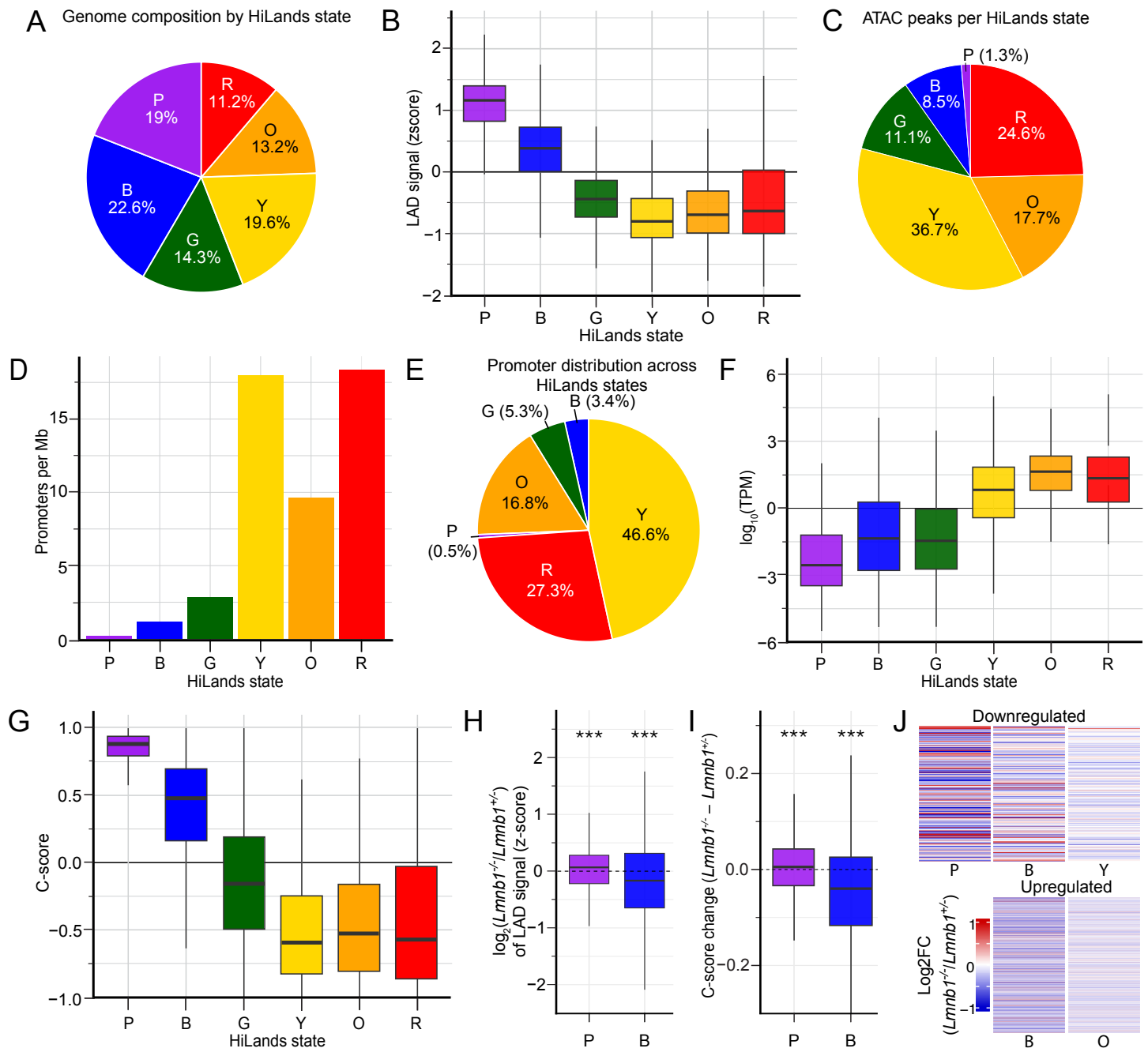

Supplement: 1 — Figure S1. Postnatal upregulation of lamin-A rescues distribution of NPC and LINC complex in Lmnb1−/− but not Lmna+/−; Lmnb1−/− cardiomyocytes. (A, B) Representative confocal images of heart tissue sections immunostained for the nuclear pore complex component TPR (A, magenta) or the LINC complex component Nesprin-1 (B, magenta) and DAPI (cyan) at E13.5 (left panels) and P7 (center and right panels). The scale bars are as indicated. N= 3 biological replicates per stain and genotype. Figure S2. Loss of lamin-B1 promotes astral microtubule arrays and disrupts the perinuclear microtubule cage in E13.5 cardiomyocytes. (A,B) Representative confocal images of cultured E13.5 cardiomyocytes immunostained for α-tubulin (α-tub, white), DAPI (cyan), and either the NPC marker TPR (magenta) (A) or the LINC complex component Sun1 (magenta) (B) across the indicated genotypes. Scale bar, 10 μm. (C) Stacked bar plot showing quantification of microtubule organization categories in E13.5 cardiomyocytes across the indicated genotypes. Bars represent the percentage of cardiomyocytes displaying each microtubule phenotype: strong perinuclear ring (black), astral array with no strong ring (blue), or polarized astral array (yellow). Data represents mean and n= 3 biological replicates. Figure S3. Bulk RNA-seq of P7 Lmnb1−/− hearts reveals downregulation of cardiomyocyte maturation genes and upregulation of cell cycle genes. (A) Volcano plot of differentially expressed genes in Lmnb1−/− versus Lmnb1+/− P7 whole hearts by bulk RNA-seq. Downregulated genes and upregulated genes are shown in blue and red, respectively. The dashed lines indicate significance thresholds (|Log2FC| > 0.26; p < 0.05). (B, C) Selected GO-terms enriched among downregulated (B) and upregulated (C) genes. Figure S4. Identification of major cell populations in the heart and characterization of ventricular cardiomyocyte clusters. (A) UMAPs of the integrated scRNA-seq dataset showing major cardiac cell populations, displayed [file NIHPP2026.05.20.726565V2-supplement-1.pdf]
